# The psychosocial adaptation of patients with skin disease: a scoping review

**DOI:** 10.1186/s12889-019-7775-0

**Published:** 2019-10-29

**Authors:** Xiu-jie Zhang, Ai-ping Wang, Tie-ying Shi, Jun Zhang, Hui Xu, Da-qiu Wang, Li Feng

**Affiliations:** 1 Department of Nursing, The First affiliated Hospital of China Medical University, Shenyang, Liaoning Province China; 2 Department of dermatology, The First affiliated Hospital of Dalian Medical University, Dalian, Liaoning Province China

**Keywords:** Skin disease, Psychosocial, Adaptation, Factors, Scoping review

## Abstract

**Background:**

Skin disease is a global public health problem that often has physiological, psychological and social impacts. However, it is not very clear how to adapt to these impacts, especially psychosocial adaptation of patients with skin disease.

**Methods:**

We searched EMBASE, PubMed, CINAHL and PsycINFO from 2009 to 2018. The following themes were extracted from the included articles: the concepts, related factors, and interventions for psychosocial adaptation of patients with skin disease. Two reviewers independently screened and analyzed.

**Results:**

From 2261 initial records, 69 studies were identified and analyzed. The concept of psychosocial adaptation in patients with skin disease was referred to under an assortment of descriptions. The related factors for psychosocial adaptation in patients with skin disease included the following: demographic factors (sex, age, education level, ethnicity, BMI, sleep quality, marital status, exercise amount, family history, the use of topical treatment only, personality and history of smoking); disease-related factors (disease severity, clinical symptoms, localization and duration); psychological factors (anxiety/depression, self-esteem, body image, stigma and suicidal ideation); and social factors (social support, social interaction, sexual life, economic burden and social acceptance). Despite being limited in quantity, several studies have clarified the benefits of adjuvant care in the form of cognitive behavioral training, educational training and self-help programs, all of which have become common methods for dealing with the psychosocial impacts.

**Conclusions:**

Based on the previous literatures, we constructed a protocol of care model for psychosocial adaptation in patients with skin disease. It not only provided the direction for developing new instruments that could assess psychosocial adaptation statue, but also a basis for helping patients adjust to changes in skin disease.

## Introduction

As the largest organ of the human body, the skin is the main barrier that resists the outside world.^1^ Because skin diseases are often not life-threatening, attention and funds may be invested in diseases considered more serious. However, the psychosocial and occupational impact of skin disease is frequently comparable to, if not greater than, other chronic medical conditions.^2^ The lifetime prevalence of skin disease was reported from European five countries, with skin disease including eczema (14.2%), atopic dermatitis (7.9%), psoriasis (5.2%) and vitiligo (1.9%).^3^ With the deterioration of environment and various pressures, the incidence of skin disease has increased in recent years. It has become a global public health problem.^4^ Many skin diseases have a chronic and repeated process, which requires us to treat the disease and help patients positive adaptation.^5^

Roy defines adaptation as the process and outcome whereby thinking and feeling persons as individuals or in groups use conscious awareness and choice to create human and environmental integration, including physiological, psychological and social aspects.^6^The British Association of Dermatologists suggested that 85% of patients with skin disease have reported that the psychosocial impacts of their disease are a major component of illness, which is a concerning statistic.^7^ Psychological and social analyses reveal that if the body is stimulated by stress and the external environment, the emotional state will change as an instinctive response.^8^ Skin disorders can significantly affect the psyche, and the psyche can significantly affect skin disorders through psycho-neuro-immuno-endocrine and behavioral mechanisms.^9^ And the stress is related to functional and psychological processes in skin disease patients with high levels of anxiety sensitivity.^8^ In response to the environmental pressures of extreme grief and fear, individuals will experience continuous tension.^10^ Skin diseases distort body image, which may have a negative impact on the psychosocial health and quality of life (QOL) of patients.^11^ A high severity of itching, pain, and scaling in psoriasis patients is related to high disease severity and low QOL and work productivity.^12^ The psychosocial adjustment to vitiligo is mainly affected by subjective factors.^13^

Therefore, it will be limited to attempts to understand the psychosocial impacts of psoriasis from the perspective of current measurements of demographic characteristics and disease severity.^14^ It is imperative to develop appropriate psychosocial adaptation (PA) evaluation tools for patients with skin disease.^15^ Various clinic models have been described to provide specialised psychodermatology care in specific settings.^16^ However, it is not clear the concepts, related factors and interventions of PA for patients with skin diseases. They were described by this scoping review. Based on the previous literatures, we attempted to present a protocol of care model for PA in patients with skin disease.

## Methods

A scoping review can examine and clarify broader areas than a systematic review to identify gaps in the evidence, clarify key concepts, and report on the types of evidence that address and inform practices in the topic area.^17^ Therefore, a scoping review method was chosen to allow for the inclusion of different study designs; this type of study follows the methodology model proposed by Arksey and O’Malley to map the various concepts underpinning this research area, as well as to clarify the related factors and interventions.^18^ We followed the guidelines of the PRISMA-ScR^19^ which is included as an Additional file 1 document to this paper. We did not provide detailed critical appraisal of individual studies or meta-analyses as this is a developing area of research. The steps of the review are outlined below.

### Identifying the research questions

This scoping review aimed to identify the various concepts and related factors of PA for patients with skin disease by mapping the existing literature in the field to provide a basis for developing instruments to assess the status of PA. Additionally, mapping showed a variety of interventions.

### Identifying relevant studies

The search strategy was formed by the project team and consulting with information specialists (see Additional file 2). The following databases EMBASE, PubMed, CINAHL and PsycINFO were chosen and searched from 2009 to 2018 for publications with no limit on language, which covered a wide range of subjects including medicine, psychosociology and nursing. EndNote was applied to exclude duplicate records and manage inclusion literatures.

### Selecting the literature

The inclusion criteria were as follows:

- **Population**: Patients experiencing skin diseases diagnosed as psoriasis, atopic dermatitis, eczema, vitiligo or chronic urticaria.
- **Range of concepts**: The psychosocial adaptation of patients in different skin conditions. According to previous research and team discussion, the following concepts were often used to reflect psychosocial impacts of patients with skin diseases: anxiety/depression, body image, stigma, self-esteem, social support, family function, financial costs and work. Some studies even equated the PA of patients with the QOL.
- **Context:** Adult population for 18 years old or older.

All articles provided primary data on the various concepts, related factors and interventions of PA for patients with skin disease from 2009 to 2018. Single case reports and comments were excluded. Firstly, in order to avoid missing valuable literature, two researchers conducted three rounds of assessments that included reading the study titles and abstracts for the inclusion and exclusion criteria. Second, the full texts of the studies identified through screening were independently assessed for eligibility by two authors. Third, the studies were classified for mapping according to the definitions and descriptions of methods provided in the publication.^17^ Finally, data extraction was undertaken by one author (JBI systematic review researcher) using a structured form. The accuracy of data extracted from the included studies was checked by another author. Any disagreements were resolved by a larger team discussion.

### Charting the data

A total of 69 articles were finally included in this review and were then subjected to data charting. The data charting took the following information into consideration: author(s), year of publication, country of origin, study population, sample size, methodology, concept, assessment tool, related factors and interventions of PA for patients with skin diseases.

### Collating, summarizing, and reporting the literature

The various concepts of PA for patients with skin diseases were identified. The related factors in the papers reviewed were classified as demographic, physiological, psychological or social factors. The interventions were reported.

## Results

**Fig. 1:**
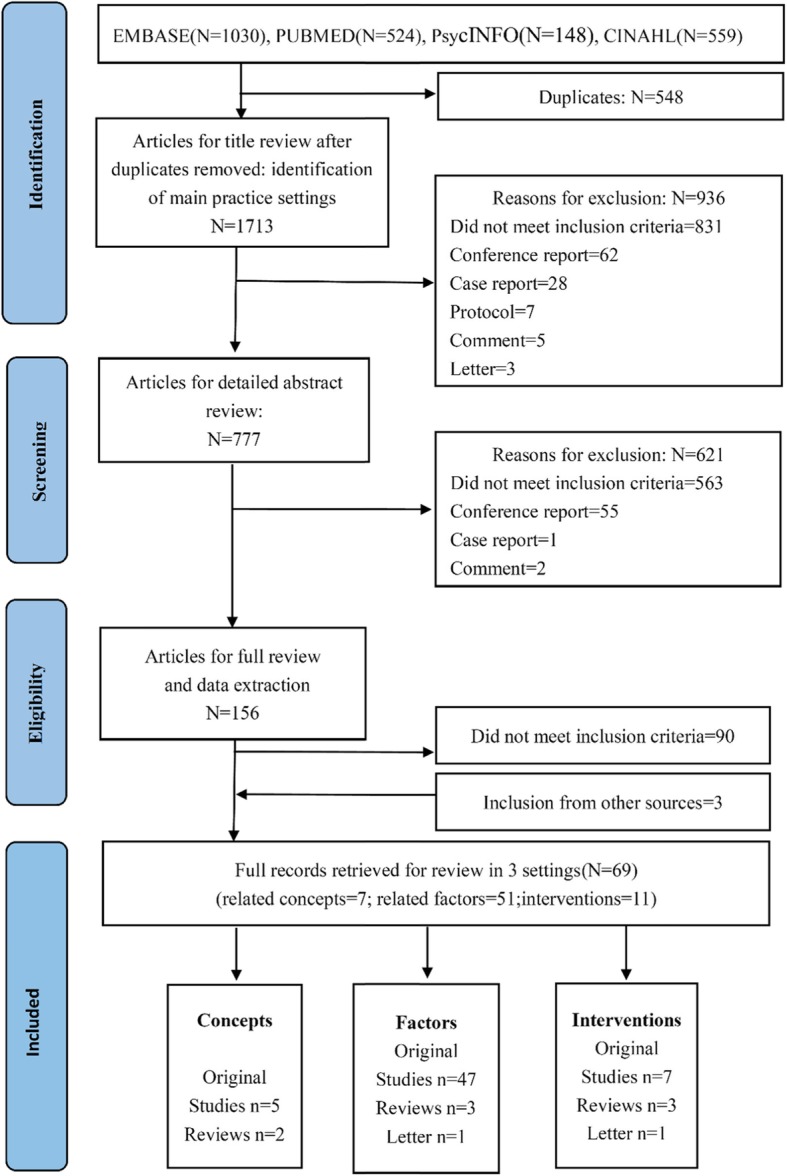
PRISMA flow diagram of illustrating literature search and selection

The search strategy yielded 2261 potential papers. After removing duplications (*n* = 548) and eliminating 936 by a first pass through the titles and abstracts, the potentially relevant literature was screened in two rounds and resulted in 69 studies. The remaining studies were clustered in the following three facets: i) various concepts of PA (*n* = 7), ii) related factors of PA (*n* = 51), and iii) interventions (*n* = 11) (Fig. 1). The characteristics of the included literature are presented in Table 1.

**Table 1:** Mapping of study characteristics of all studies included in this review

Study characteristics (*N* = 69)	N (%)
Publication years	
2009–2013	16 (23.19)
2014-June 2018	53 (76.81)
Country of origin	
UK	8 (11.59)
USA	8 (11.59)
Poland	8 (11.59)
India	6 (8.70)
German	5 (7.25)
China	4 (5.80)
Japan	4 (5.80)
Denmark	2 (2.90)
Korea	2 (2.90)
Italy	2 (2.90)
Greece	2 (2.90)
Malaysia	2 (2.90)
Canada	2 (2.90)
Spain	2 (2.90)
Singapore	2 (2.90)
Portugal	2 (2.90)
Sweden	2 (2.90)
Netherlands	1 (1.45)
Turkey	1 (1.45)
Iran	1 (1.45)
Finland	1 (1.45)
Ireland	1 (1.45)
Egypt	1 (1.45)
Study types	
Cross-sectional study	43 (62.32)
Randomized controlled trial	5 (7.25)
Review	5 (7.25)
Systematic review	3 (4.35)
Case-control study	2 (2.90)
Prospective cross-sectional study	2 (2.90)
Case series study	2 (2.90)
Experimental design	2 (2.90)
Qualitative study	2 (2.90)
Letter	2 (2.90)
Develop a rating scale	1 (1.45)
Study population	
Psoriasis	44 (63.77)
Atopic dermatitis	10 (14.49)
Vitiligo	9 (13.04)
Eczema	3 (4.35)
Skin disease	3 (4.35)

### Various concepts of psychosocial adaptation for patients with skin disease

A clear conceptual definition of psychosocial adaptation is identified by Rodgers’ evolutionary concept analysis, and the identified attributes of PA include change, process, continuity, interaction and influence, all of which were present in the multidisciplinary literature reviewed, thus demonstrating the wide use of the concept.^20, 21^ In the nineteenth century, skin diseases were linked to psychosocial factors. The mechanism was proposed and clarified in subsequent decades, and multidisciplinary collaboration was crucial to promote the adaptation of patients with skin diseases.^22^ PA was referred to under an assortment of descriptions in skin diseases including psychosocial factor,^11, 23^ burden,^24, 25^ impact,^26^ morbidity,^15^ and aspect.^27^ The measurement methods used in the literature are shown in Table 2.

**Table 2:** Measurement methods used in the literatures

Domain	Related concept	Measurement methods (N: number of studies reporting)
psychosocial	Quality of life	Dermatology Life Quality Index (DLQI) (*N* = 32) European Quality of Life-5 Dimensions (EQ-5D) (*N* = 7) Short Form Health Survey (SF-36) (*N* = 2) General Health Questionnaire (GHQ) (*N* = 1) World Health Organization Quality of Life-BREF (*N* = 1) Short form of the General Health Questionnaire (GHQ-28) (*N* = 1) Short-Form 12 health status instrument (*N* = 1) Eczema Quality of Life Scale (EQOLS) (*N* = 1) Revised Dyadic Adjustment Scale (R-DAS) (*N* = 1)
	Subjective Burden	Skindex-29 (*N* = 2) Skindex-16 (*N* = 2) Skindex-19 (*N* = 1)
psychological	Anxiety and depression	Hospital Anxiety and Depression Scale (HADS) (*N* = 13) Beck Depression Inventory (BDI) (*N* = 8) 42-item Depression, Anxiety and Stress Scale (DASS-42) (*N* = 2) Social Anxiety Questionnaire (SAQ) (*N* = 2) Center for Epidemiologic Studies Depression Scale (CES-D) (*N* = 2) Self-Rating Depression Scale (SDS)&Self-Rating Anxiety (SAS) (*N* = 1) Patient Health Questionnaire Depression Scale (PHQ-9) (*N* = 1) Generalized Anxiety Disorder Scale (GAD-7) (*N* = 1) Arabic version of the Depression, Anxiety and Stress Scale (*N* = 1) Penn State Worry Questionnaire (*N* = 1) State-Trait-Anxiety Inventory (*N* = 1)
	Body image	Body Emotions Scale (BES) (*N* = 2) Appearance Schemas Inventory-Revised (ASI-R) (*N* = 2) Body Image Scale (BIS) (*N* = 2) Body Dysmorphic Disorder Questionnaire (BDDQ) (*N* = 1) Female Genital Self-image Score (FGSIS) (*N* = 1) Acceptance of Life with the Disease Scale (ALDS) (*N* = 1) Perceived Health Status (PHS) (*N* = 1) Multidimensional Body-Self Relations Questionnaire (MBSRQ) (*N* = 1) Skin Satisfaction Questionnaire (SSQ) (*N* = 1) Derriford Appearance Scale (DAS-24) (*N* = 1)
	Self-esteem	Rosenberg Self-esteem Scale (RSES) (*N* = 5) Fears of Compassion Scales (FCS) (*N* = 1) Brief Fear of Negative Evaluation Scale (Brief FNE) (*N* = 1)
	Stigmatization	Stigmatization Scale (*N* = 3) Stigmatization and Psoriasis Questionnaire (SPQ) (*N* = 1) Psoriasis disease stigma questionnaire (PSQ) (*N* = 1) Internalized Stigma Scale (ISS) (*N* = 1)
	Alexithymia	Toronto Alexithymia Scale (TAS) (*N* = 1)
social	Social support	Berlin Social Support Scales (BSSS) (*N* = 1) Social support rating scale (SSS) (*N* = 1) Disease-Related Social Support Scale (DRSS) (*N* = 1) Multidimensional Scale of Perceived Social Support (MSPSS) (*N* = 1)
	Social interaction	Social Readjustment Rating Scale (*N* = 1) Participation Scale Questionnaire (*N* = 1)
	Social acceptance	Marlowe–Crowne Social Desirability Scale (MC-SDS) (*N* = 1)
	Occupational effect	Work Productivity and Activity Impairment (WPAI) (*N* = 4) Work Productivity and Activity Impairment Questionnaire: Psoriasis (WPAI-PSO) (*N* = 1) Work Productivity and Activity Impairment-General Health (WPAI-GH) (*N* = 1) Work Limitation Questionnaire (WLQ) (*N* = 1) Total work productivity impairment (TWPI) (*N* = 1) Total activity impairment (TAI) (*N* = 1)
	Economic burden	Direct costs (*N* = 1) Direct Costs of AD: medications, over-the-counter costs, medical testing and procedure expenses, physician visits, out-of-pocket expenses, transportation, and childcare increased as severity of AD worsened (*N* = 1) Indirect Costs of AD (productivity loss of caregivers) (*N* = 1) Lost productivity (*N* = 1)
Physical	Disease severity	Psoriasis Area and Severity Index (PASI) (*N* = 16) Psoriasis Disability Index (PDI) (*N* = 3) Vitiligo area scoring index (VASI) (*N* = 1) Distress Thermometer (DT) (*N* = 1) Physician global assessment (PGA) (*N* = 1) Severity scoring of AD (SCORAD) (*N* = 1) Physicians Global Assessment (PGA) score (*N* = 1) Hand eczema severity:10-point visual analogue scale (VAS) (*N* = 1) Patient-Oriented Scoring Atopic Dermatitis (PO-SCORAD) index (*N* = 1) Impact of chronic Skin Disease on Daily Life (ISDL) (*N* = 1) Self-Administered Psoriasis Area and Severity Index (SAPASI) (*N* = 1)
	Clinical feature	Itching/pruritus: Visual Analogue Scale (VAS) (*N* = 2) Juckreiz-Kognitions-Fragebogen questionnaire (*N* = 1) SCORAD index (N = 1)
	Distribution and extent	Body Surface Area (BSA) (*N* = 6) Body surface involvement (*N* = 1)
Others	Sexual life	Massachusetts General Hospital-Sexual Functioning Questionnaire (MGH-SFQ) (*N* = 2) Female Sexual Function Index (FSFI) (*N* = 1) International Index of Erectile Function (IIEF) (*N* = 1)
	Stress	Psoriasis Life Stress Inventory (PLSI) (*N* = 1) Stress Self-assessment Scale (*N* = 1)
	Personality	Eysenck Personality Questionnaire (EPQ) (*N* = 1) Eysenck Personality Inventory (*N* = 1)
	Life course	Course of life questionnaire (COLQ) (*N* = 1) Modified psoriasis life stress inventory (*N* = 1)
	Loneliness	UCLA loneliness scale (UCLA-Version 3) (*N* = 1)
	Sleep Quality	Medical Outcomes Study Sleep Scale (MOS-SS) (*N* = 1)
	Disease control	Urticaria Control Test (UCT) (*N* = 1)
	Knowledge	Psoriasis Knowledge Assessment Questionnaire (*N* = 1)
	Biological Markers	CRP and cytokines IL-1β, IL-6, TNFα, and IL-17 (*N* = 1)
	Mindfulness	Five Facet Mindfulness Questionnaire (*N* = 1)

### Related factors of psychosocial adaptation for patients with skin disease

Table 3 shows the related factors of PA for patients with skin disease including the demographic, disease-related, psychological and social factors.

**Table 3:** related factors of psychosocial adaptation in patients with skin disease

Author, year	Study population and sample size	Research topic	Demographic factors	Disease related factors	Psychological factors	Social factors	Others
Nayak et al.(2018)	Psoriasis(*n* = 102)	Quality of Life		Disease severity (−)*		Family income (+)*	
Kwan et al.(2018)	Psoriasis(*n* = 102)	Quality of life	Age (+)*	Disease severity (−)*	Depression/Anxiety (−)*		
Itakura et al.(2018)	Chronic urticaria, AD, psoriasis (*n* = 1443, 1668,435)	Quality of life				Work productivity (+)*	
Lee et al.(2018)	Atopic Dermatitis (*n* = 677)	Quality of life			Depression (−)* Suicidal ideation (−)*		Sleep disturbance (+)*
Bidaki et al.(2018)	Vitiligo (*n* = 126)	Social acceptance	Woman* Marital status*	Lesions distribution (face and neck) * Disease duration less than 5 years*			
Hebert et al.(2018)	Atopic dermatitis (*n* = 76)	Economic burden		Disease serverity (+)* Itching (+)*		Lost productivity Outpatient expense	
Norreslet et al.(2018)	Atopic dermatitis (*n* = 23)	Work life				Job choice Change or loss of job Disability pension	
Lakuta et al.(2018)	Psoriasis (*n* = 193)	Stigmatization Depression Social anxiety		Location and extent of psoriasis*			
Kwak et al.(2017)	Atopic dermatitis (*n* = 662)	Occupational characteristics				Work format/hours Job classification Employment status	
Kwan et al.(2017)	Psoriasis (n = 102)	Quality of life	India ethnicity Education (−)*		Depression (−)*	Employ status Monthly income (+)*	
Nazik et al.(2017)	Psoriasis (*n* = 92VS98)	Quality of life		Disease severity (−)*	Self esteem (+)* Body image (−)*		
Alpsoy et al.(2017)	Psoriasis (*n* = 1485)	Internalized stigma	Education (−)* Family history*	Disease severity (+)* Lesions distribution (visible parts of body) * Disease duration (+)*		Income level (−)*	Quality of life (−)*
Dieris-Hirche et al.(2017)	Atopic dermatitis (*n* = 181)	Suicidality	Age (−)*	Disease severity (+)*	Depression/Anxiety (+)*	Family support (−)*	
Lakuta et al.(2017)	Psoriasis (*n* = 148)	Depressive	Female*	Disease duration (−)*	Stigma (+)*	Social support (−)*	
Rosinska et al.(2017)	Psoriasis (*n* = 54)	Depressive	Female*		Body image (−)*		
Nicholas et al.(2017)	Atopic Dermatitis	depression and suicidality	Female* Age (+)*	Disease serverity (−)*			
Lamb et al.(2017)	Psoriasis (*n* = 607)	Anxiety and Depression	Female* Asian ethnicity* Topical treatment only*	Disease serverity (+)*			
Lakuta et al.(2017)	Psoriasis (*n* = 193)	Social anxiety and depression	Female*	Disease serverity (+)* Disease duration (+)*			
Geale et al.(2017)	Psoriasis (*n* = 2674)	Quality of life		Disease severity (−)*			
Lesner et al.(2017)	Psoriasis (*n* = 682)	Quality of life	Marital status*	Itch intensity*	Depression/Anxiety* Suicidal ideation*		
Kimball et al.(2016)	Psoriasis (*n* = 694)	Work productivity	Sleep problems*	Pruritus (−)*			
Zhu et al.(2016)	Psoriasis (*n* = 108)	Stigma				Social support (−)* Social interaction (−)*	Quality of life (−)*
Sarhan et al.(2016)	Vitiligo (*n* = 50VS25)	Quality of life		Lesions distribution*			Sexual life*
Korman et al.(2016)	Psoriasis (*n* = 694)	Quality of life		Disease severity (−)* Scaling, itching, pain*		Work productivity (+)*	
Bonotis et al.(2016)	Vitiligo (*n* = 216)	Quality of life	Sex* Personality*		Self esteem (−)*		
Cazzaniga et al.(2016)	Chronic hand eczema (*n* = 199)	Quality of life Psychosocial adjustment				Job loss and change	High rate of sick leave Intense use of health care services
Tee et al.(2016)	Psoriasis (*n* = 100)	Quality of life		Disease severity (−)*	Depression/Anxiety (−)*		
Molina-Leyva et al.(2016)	Psoriasis (*n* = 79VS79)	Erectile Dysfunction	Smoking BMI		Depression/Anxiety (−)*		
Ji et al.(2016)	Psoriasis (*n* = 191VS191)	Erectile Dysfunction	Age* Hypertention Hyperlipidemia*		Depression*		
Innamorati et al.(2016)	Psoriasis (*n* = 100VS97)	Quality of life	BMI*		Depression*		
Korman et al.(2016)	Psoriasis (*n* = 681)	Quality of life		Disease severity (−)*		Work productivity (+)*	
Molina-Leyva et al. (2015)	Psoriasis (*n* = 133)	Sexual Function		Distribution of lesions*			
Ahmed et al.(2015)	hand eczema (*n* = 1023)	Self-esteem					
Korman et al.(2015)	Psoriasis (*n* = 700)	Quality of life		Disease severity (−)* Scaling, itching, pain*		Work productivity (+)*	
Schmitt et al.(2015)	Psoriasis (*n* = 201)	Quality of life				Work productivity Indirect costs	
Ayala et al.(2014)	Psoriasis (*n* = 787)	Work-related problem	Sex* Low education*	Disease serverity (+)* Localization*	Shame* Anger* Self-esteem*		
Khoury et al.(2014)	Psoriasis (*n* = 8)	Body image	Exercise (+)*	Body coverage (−)*		Social support (+)*	Sexual inhibitions (+)*
Mattila et al. (2013)	Psoriasis (*n* = 262)	Work				Change of occupation Sick leave days Early retirement from work	
Yano et al. (2013)	Atopic Dermatitis (*n* = 112)	Work productivity and activity impairment		Disease serverity (+)*			Quality of life (+)*
Chen et al. (2013)	Psoriasis (*n* = 12,300VS61,500)	Sexual dysfunction	Male* Aged*				
Lewis-Beck et al. (2013)	Psoriasis (*n* = 199)	Quality of life		Itching intensity (−)* Pain (−)* Scaling (−)*		Work productivity (+)*	
Chrostowska-Plak et al. (2013)	Atopic Dermatitis (*n* = 89)	Quality of life		Pruritus (−)*	Depression (−)*		
Schneider et al. (2013)	Psoriasis (*n* = 50)	Social anxiety Social avoidance		Disease severity (+)*	Feelings of helplessness (+)*	Social support (−)*	Quality of life (−)*
Premkumar et al. (2013)	Psoriasis (*n* = 300)	Quality of life	Aged* Low education*	Disease severity (−)*		Stigma (−)*	
Sampogna et al. (2012)	Psoriasis (*n* = 936)	Quality of life	Female* Low education*	Disease severity (−)*	Depression/Anxiety (−)*	Shame, angry and problems in daily activities and social life	
Janowski et al. (2012)	Psoriasis (*n* = 113)	Quality of life /Adaptation	Gender*			Social support (+)*	
Levy et al.(2012)	Psoriasis (*n* = 90)	Quality of life				Direct costs* Lose productivity*	Economy burden
Chan et al. (2012)	Vitiligo (*n* = 145)	Depression	Age (+)* Sex*		Self-esteem (+)*		
Brito et al. (2012)	Psoriasis (*n* = 101patients + 78 partners)	Adjustment			Body image (+)*	Relationship between patients and partners	
Pereira et al. (2012)	Psoriasis (*n* = 101)	Adjustment		Disease severity (−)*	Depression/Anxiety (−)*	Family coping in patients and their partners	
Pichaimuthu et al. (2011)	Vitiligo and psoriasis (*n* = 300)	Stigma				Participant restrictions (+)*	

**Note:** + positive correlation, −negative correlation, *p < 0.05 statistically significant

### Demographic factors

With regard to demographic facets, the key factors reported were sex,^13, 28–38^ age,^31, 35, 38–41^ education level,^34, 36, 41–43^ ethnicity,^32, 42^ BMI,^44, 45^ sleep quality,^46, 47^ marital status,^28, 48^ exercise amount,^49^ family history,^43^ the use of topical treatment only,^32^ personality^13^ and history of smoking.^44^ Females were more prone to depressive and psychosocial maladaptation than males with skin disease.^28, 31, 50^ Because females were more likely to believe in the importance of physical appearance to their personal or social values than males, their investment in physical attractiveness was significantly increased. Psychological impacts related to skin disease may largely be attributed to the patients’ maladaptive assumptions about appearance and society’s focus on the perfect body and beauty. However, the genital lesions in males were more prone to cause sexual dysfunction than the lesions in females.^35^ There was no agreement for the impact of age on psychosocial level.^31^ Younger psoriasis patients can experience feelings of embarrassment, disturbance of daily activities, poor physical health, and low productivity at work. Nevertheless, it was also found that old age was related to a high risk for depression in atopic dermatitis patients. Education level also influenced the QOL of patients with psoriasis.

### Disease-related factors

The disease-related factors were severity,^12, 31–34, 36, 39–41, 43, 49, 51–60^ clinical symptoms (itching,^47, 48, 52, 61^ pain, scaling),^12, 54, 62^ localization (visible and genital parts)^28, 34, 43, 50, 63, 64^ and duration.^28, 29, 33, 43^ The severity of the skin disease was associated with the level of depression and anxiety, and it had a negative effect on QOL.^23, 27^ Itching is the cardinal clinical symptom of patients with skin disease, which can result in sleep deprivation and mental disorders. However, the localization of the skin lesions was often more important than the disease severity and was associated with negative mental health, including depression, social anxiety, self-image disorder, and stigmatization. The ‘sensitive’ body regions were defined as the visible parts of the body, which included the scalp, face, neck, hand and fingernails.^43^ Additionally, the psoriasis lesions located on the genitals, buttocks, abdomen, chest or lumbar region were more likely to lead to sexual dysfunction.^64^ The clinical symptoms of psoriasis, particularly itching, pain and scaling, negatively affected health outcomes and work productivity.^62^

### Psychological factors

With respect to psychological facet, the related factors included anxiety and depression,^36, 39, 40, 42, 44–46, 48, 55, 60, 61, 65^ self-esteem,^13, 34, 53, 66^ body image,^30, 53, 67^ stigma^29, 41^ and suicidal ideation.^46, 48^ Skin disease patients have a high level of anxiety or depression. Proinflammatory cytokines such as IL-1 and IL-6 were found in both psoriasis and depression, indicating that the inflammatory process may be involved in the progression of both diseases.^68^ Depression in psoriasis patients was related to a high risk of stroke and cardiovascular death, especially during acute depression.^69^ The adaptation of vitiligo patients has been considered to be affected by self-esteem levels. The following five common themes of stigma have been identified in patients with psoriasis: anticipation of rejection, feelings of being flawed, sensitivity to the attitudes of society, secretiveness, guilt and shame.^15^ A high level of stigma and low self-esteem have negative effects on patient compliance.

### Social factors

The social factors of PA in patients with skin disease were: social support,^29, 37, 40, 49, 57, 70^ social interaction,^36, 67, 70, 71^ sexual life^49, 63^ and economic burden ^42, 72–76^ (medical expenses,^52^ work productivity,^12, 52, 54, 59, 62, 77, 78^ income level^43, 51^). It was found that high levels of perceived social support were positively correlated with the low occurrence of depressive symptoms.^11^ The marriages and relationships of 50% of vitiligo patients were negatively affected by skin disease.^79^ Due to its physical symptoms and the stigma caused by the appearance of skin, psoriasis can be considered a socially isolating disease.^68^ Psoriasis, a chronic inflammatory skin disease, seems to be related to erectile dysfunction, which was a predictor of future cardiovascular disease.^65^ It is critical to accurately evaluate effective treatments of skin disease to understand the interaction between lost productivity, direct costs and quality of life.^76^

### Interventions of psychosocial adaptation for patients with skin disease

The outcomes of PA include positive and negative aspects.^20^ Table 4 shows the PA interventions of skin disease included cognitive behavioral therapy,^80–86^ educational training,^82, 87–89^ self-help programs,^80, 81, 84^ psychotherapy^84^ and communication.^90^

**Table 4:** Psychosocial adaptation interventions of skin disease

Author, year	Study population (sample size)	Type of Intervention	Follow up	Outcome
Rzepecki et al.(2018)	Vitiligo	Adjuvant care: group therapy cognitive behavioral therapy self-help programs		
Nagarajan et al.(2018)	Psoriasis and control,(*n* = 52VS52)	Video-assisted teaching program regarding psoriasis on the level of knowledge and relaxation therapy	3 months	Improving the knowledge and quality of life of patients with psoriasis.
Keyworth et al.(2018)	Psoriasis (*n* = 217)	Health risk communication: message framing theory Gain-frame messages and loss-frame messages		Alcohol reduction: loss-framed messages appear to be more effective for cardiovascular disease risk reduction information. Psoriasis symptom reduction: gain-framed messages are more effective. Messages about cardiovascular disease result in higher emotional responses compared to messages about psoriasis symptom reduction.
Paul et al.(2018)	Psoriasis (*n* = 94)	Mindfulness-based cognitive therapy (MBCT) (*n* = 25) Mindfulness-based self-compassion therapy (MBSCT) (*n* = 25) Self-help MBSCT (MBSCT-SH) (*n* = 22) Treatment-as-usual (TAU) (*n* = 22)	12 months	Improving the long-term psychological and physical outcomes of individuals with psoriasis.
Zill et al.(2018)	Psoriasis	Cognitive behavioral techniques: Mindfulness and meditation Emotional writing Individual and group setting		Most studies reported positive but nonsignificant effects on the different patient-reported outcomes.
Heratizadeh et al.(2017)	Atopic dermatitis intervention group (*n* = 168) intervention group (*n* = 168)	Educational training: a comprehensive 12-h training manual	12 months	Itching (catastrophizing cognitions):Juckreiz- Kognitions -Fragebogen questionnaire Social anxiety: Marburger Hautfragebogen questionnaire Subjective burden by symptoms of the disease: Skindex-29 questionnaire Improvement of disease signs and symptoms: SCORAD index
Hashimoto et al.(2017)	Atopic dermatitis (*n* = 12)	Psychological and educational interventions: The psychological interventions included supportive, cognitive, behavioral, and psychodynamic psychotherapies, cognitive-behavioral therapy, and physical training such as progressive muscle relaxation. Lectures, audiotapes, books, videotapes, and question-and-answer sessions for the educational interventions contained information on the disease, treatment options, and strategies for management and prevention.		The data did not have sufficient power to provide evidence-based conclusions.
Van et al. (2016)	Psoriasis care as usual (CAU, *n* = 66) (ICBT+ CAU (*n* = 65)	Internet-based cognitive behavioral therapy (ICBT)	6 months	Results underline the promise of therapist-guided, individually tailored ICBT to improve physical functioning and reduce the impact of psoriasis on daily activities in patients with a psychological risk profile. Establishing a good therapeutic relationship early on may be an important factor that influences treatment outcomes in personalized ICBT interventions. Further research is needed to evaluate ICBT effectiveness in additional samples and to explore its underlying mechanisms.
Jha et al.(2016)	Vitiligo (*n* = 13)	Behavior therapy techniques: Psycho-education Breathing/relaxation and imagery Self-statements Exposure and desensitization	3 months	The feasibility of such therapy would depend upon the willingness and ability of both the dermatologist and the patient to set aside the time required. Interventions with less frequent sessions of shorter duration may ensure better patient compliance.
Shah et al.(2014)	Vitiligo CBSH+(*n* = 24) CBSH (*n* = 25) Control (*n* = 26)	Cognitive behavioral self-help intervention (CBSH) had three parts: Psycho-education, including a description of how social anxiety is likely to be maintained in vitiligo; Symptom monitoring with an emphasis on the recognition of self-focused attention and triggers of anxiety; Guided imagery based relaxation and techniques for switching attention.	2 months	The findings demonstrate that augmented CBSH provides a relatively simple and accessible intervention that can result in a clinically significant reduction in social anxiety. The augmented intervention has potential and might be further developed and evaluated in subsequent trials.
Bundy et al.(2013)	Psoriasis eTIPs (*n* = 67) Control (*n* = 68)	Electronic Cognitive behavioral therapy intervention for Psoriasis (eTIPs), 6-week programme	6 months	This first online CBT intervention for people with skin disease showed improvement in anxiety and quality of life in patients with psoriasis.

## Discussion

This scoping review analyzed the contents of 69 papers with results that were three-fold: i) some reported the various concepts of PA for patients with skin disease, which required that future research should unify the terms; ii) some reported the related factors of PA for patients with skin disease, which provided a basis for developing instruments that assess the status of PA for patients with skin disease; and iii) others reported a variety of interventions, which provided a basis for formulating a protocol of care model for PA in patients with skin disease.

Patients with skin disease often have to cope with a condition that leads to physical disfigurement, psychological destruction and social stigma. Although a large number of studies have been conducted on the treatment of patients with skin diseases, few studies have been directed towards the status and interventions of the psychosocial adaptation for patients with skin disease. It was shown that psychoeducational intervention for acceptance and managing social impact is needed, which is also the first step to informing the development of a patient-centered psychological intervention.^91^ Adding nondrug treatments such as biofeedback, cognitive behavioral methods, CES, EFT, EMDR, hypnosis, mindfulness meditation, placebo effect, or suggestions often enhances the therapeutic effect.^9^ The major routes for coping with the impacts of skin disease include the doctor-patient relationship, education of the patient and the community about the actual nature of these diseases, and more structured therapeutic strategies such as individual, group, or behavioral therapy. In response to patient feedback and NICE guidelines, the ‘Psoriasis Direct’ service was launched in 2013; this service aims to give patients open access to specialist nurses when they need it for secondary care, and ‘Psoriasis Direct’ has received overwhelmingly positive feedback.^92^ Despite being limited in quantity, several studies have clarified the benefits of adjuvant care in the form of cognitive behavioral training, educational training and self-help programs. An electronic health record system for patients with skin disease has not been established for long-term follow-up, so there is a lack of a systematic care model and financial support.^93^

**Fig. 2:**
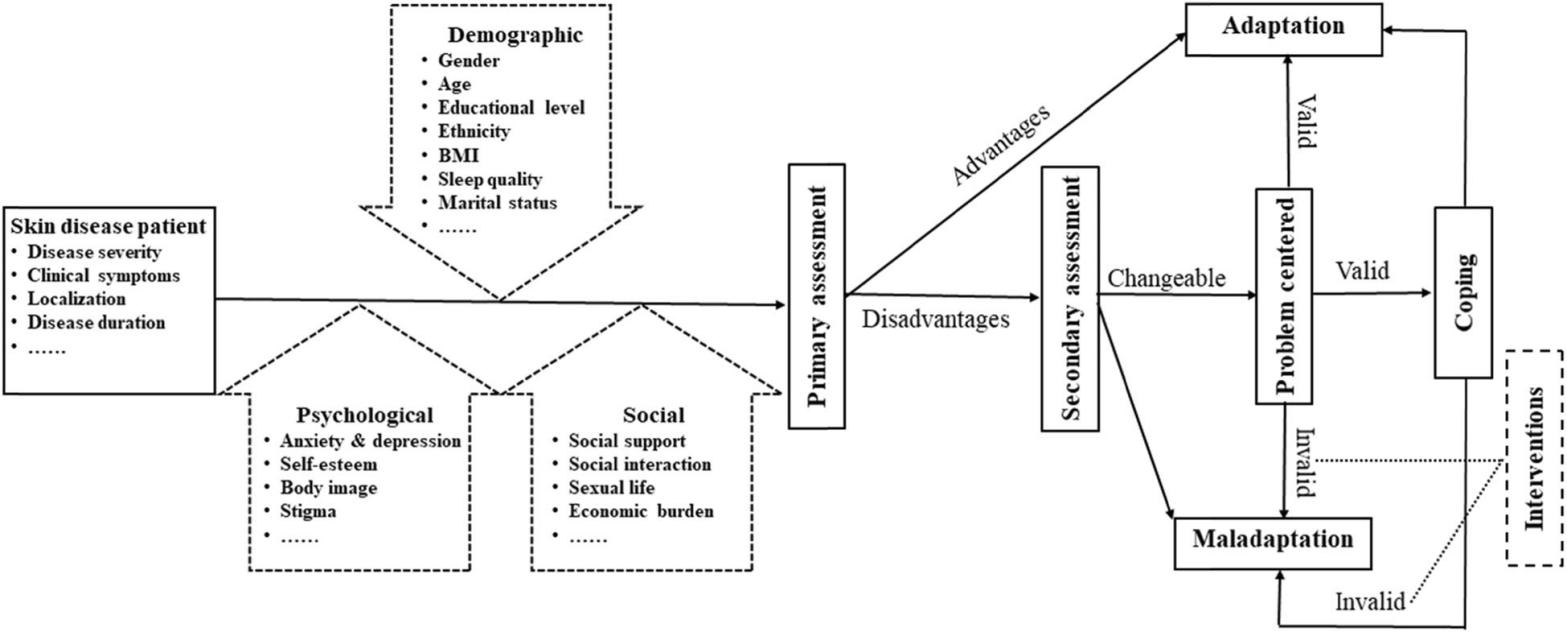
A protocol of care model for psychosocial adaptation in patients with skin disease

Most researchers have posited models in which adaptation is conceptualized as a process of change in reaction triggered by functional limitations associated with external environmental antecedents (eg, injury, accidents, traumas) or internal pathogenic condition (eg, disease).^21^ And the adaptation process suggests an unfolding paradigm in which the individual’s reactions to his or her chronic illness or disability follow a stable sequence of phase (ie, partially overlapping and nonexclusive psychosocial reactions), or stage (ie, discrete and categorically exclusive psychosocial reactions) that can be temporally and hierarchically ordered. Others view psychosocial adaptation to chronic illness and disability as one of a set of independent and nonsequential patterns of human behavior.^21^ Based on previous theories and studies, when individuals have skin diseases, the individuals will make different primary assessments due to their different demographic, psychological and social conditions. If individuals think they can cope with the skin disease, they will adopt a positive attitude and behavior to face it, which refers to positive psychosocial adaptation. However, if individuals think they cannot cope with the skin disease, they will suffer from psychosocial maladaptation or conduct a secondary assessment. The above two situations continued to occur after the secondary assessment. If we can carry out targeted psychosocial intervention before the individual experience invalid adaptation, we can help patients positively deal with the skin disease and then promote patient adaptation (Fig. 2).

### Strength and limitations

This research included studies in different settings, which brought to light the range of concept and related factors of PA for patients with skin disease, which could provide the direction for further research. A scoping review method was chosen to allow for the inclusion of different study designs, and it does not involve detailed critical appraisal of individual studies or meta-analyses. Considering partial databases selected and gray literature not included, the results are used only as an overview of the field.

## Conclusion

The clinical process of a series of skin diseases is the result of a complex and sometimes reciprocal interaction among biological, psychological, and social factors, all of which can play a role in the occurrence and development of skin diseases. This review described the range of concept and related factors of psychosocial adaptation for patients with skin disease, which could contribute to the development of new instruments. The protocol of care model based on previous theory and research could provide directions for care and policy that promote psychosocial adaptation for patients with skin disease. Further research is needed to examine the effectiveness of psychosocial interventions based on the protocol of care model for individuals with skin disease.

## Supplementary information

**Additional file 1.** Preferred Reporting Items for Systematic reviews and Meta-Analyses extension for Scoping Reviews (PRISMA-ScR) Checklist.

**Additional file 2.**

## Data Availability

All data generated or analysed during this study are included in this published article and its supplementary information files.

## Abbreviations

PA: Psychosocial Adaptation
QOL: Quality of Life
