# Supplementary material for: The psychosocial adaptation of patients with skin disease: a scoping review
Source: BMC Public Health. 2019 Oct 29;19:1404. doi: 10.1186/s12889-019-7775-0 (PMC6819547; doi:10.1186/s12889-019-7775-0)
Supplement: Supplementary file 2 — Additional file 2. [file 12889_2019_7775_MOESM2_ESM.docx]

**PubMed Search Strategy**

1 "Psoriasis"[Mesh]

2 "Dermatitis, Atopic"[Mesh]

3 "Vitiligo"[Mesh]

4 "Eczema"[Mesh]

5 Chronic urticaria. tw.

6 1 or 2 or 3 or 4 or 5

7 (psychosocial or psycholog*).tw,ti.

8 (social or sociolog*).tw,ti.

9 (financial or econom*).tw,ti.

10 (anxiety or depression or stigma or family or work* or self-esteem) .tw,ti.

11 (body image) .tw,ti.

12 (dysmorphic disorder) .tw,ti.

13 7 or 8 or 9 or 10 or 11 or 12 or 13

14 limited (Publication date from 2009/01/01 to 2018/08/31)

15 6 and 13 and 14

**CINAHL Search Strategy**

1 (Psoriasis OR Dermatitis OR Eczema OR Vitiligo OR Chronic urticaria) .tw,ti.

2 (Psychosocial OR psycholog* OR social OR sociolog* OR financial OR econom* OR anxiety OR depression OR stigma OR family OR work* OR self-esteem OR body image OR dysmorphic disorder) .tw,ab.

3 (1 AND 2) Published Date: 20090101-20180831

**PsycINFO Search Strategy**

1 (Psoriasis OR Dermatitis OR Eczema OR Vitiligo OR Chronic urticaria) .tw,ti.

2 (Psychosocial OR psycholog* OR social OR sociolog* OR financial OR econom* OR anxiety OR depression OR stigma OR family OR work* OR self-esteem OR body image OR dysmorphic disorder) .tw,ab.

3 (1 AND 2) Published Date: 2009-2018

**EMBASR Search Strategy**

1 (Psoriasis OR Dermatitis OR Eczema OR Vitiligo OR Chronic urticaria) .m_titl.

2 (Psychosocial OR psycholog* OR social OR sociolog* OR financial OR econom* OR anxiety OR depression OR stigma OR family OR work* OR self-esteem OR body image OR dysmorphic disorder) .m_titl. .

3 (1 AND 2) Published Date: 2009-2018
